# Distinct Mechanisms Underlying Resveratrol-Mediated Protection from Types of Cellular Stress in C6 Glioma Cells

**DOI:** 10.3390/ijms18071521

**Published:** 2017-07-14

**Authors:** John C. Means, Bryan C. Gerdes, Peter Koulen

**Affiliations:** 1 Vision Research Center, Department of Ophthalmology, School of Medicine, University of Missouri—Kansas City, 2411 Holmes St., Kansas City, MO 64108, USA; meanscj@umkc.edu (J.C.M.); gerdesbc@umkc.edu (B.C.G.); 2 Department of Biomedical Sciences, School of Medicine, University of Missouri—Kansas City, 2411 Holmes St., Kansas City, MO 64108, USA

**Keywords:** caspase, central nervous system, DNA damage, glia, neurofibrillary tangles, oxidative stress, tauopathy

## Abstract

The polyphenolic phytostilbene, *trans*-resveratrol, is found in high amounts in several types and tissues of plants, including grapes, and has been proposed to have beneficial effects in the central nervous system due to its activity as an antioxidant. The objective of the present study was to identify the mechanisms underlying the protective effects of resveratrol under conditions of oxidative stress or DNA damage, induced by the extracellularly applied oxidant, *tert*-butyl hydrogen peroxide, or UV-irradiation, respectively. In C6 glioma cells, a model system for glial cell biology and pharmacology, resveratrol was protective against both types of insult. Prevention of tau protein cleavage and of the formation of neurofibrillary tangles were identified as mechanisms of action of resveratrol-mediated protection in both paradigms of cellular damage. However, depending on the type of insult, resveratrol exerted its protective activity differentially: under conditions of chemically induced oxidative stress, inhibition of caspase activity, while with DNA damage, resveratrol regulated tau phosphorylation at Ser^422^. Results advance our understanding of resveratrol’s complex impact on cellular signaling pathway and contribute to the notion of resveratrol’s role as a pleiotropic therapeutic agent.

## 1. Introduction

Resveratrol (3,5,4′-trihydroxy-trans-stilbene) a polyphenol found in plant tissue, such as grapes, possesses antioxidant properties, which are being discussed as therapeutically relevant, e.g., in the context of resveratrol’s biological effects as a component of red wine [1,2]. In addition, resveratrol has been shown to have neuroprotective properties. For example, resveratrol promotes clearance of β-amyloid (Aβ) peptides, reduces oxidative stress, and reduces neuronal apoptosis [3]. Resveratrol protected rat C6 glioma cells from Aβ mediated toxicity [4]. Ulakcsai and colleagues determined that resveratrol inhibits caspase activation in primary fibroblasts after serum starvation [5]. Studies using animal models of Alzheimer’s disease (AD) suggest that resveratrol can reduce neurodegeneration, prevent impaired learning and memory formation, and prevent amyloid plaque formation [6].

In addition, resveratrol’s antioxidant properties have been successfully employed to protect cells against hydrogen peroxide-induced oxidative stress [7,8], and pretreatment with resveratrol promoted cell survival and protection against UV-irradiation induced cell death [9,10]. The goal of our study was to mechanistically dissect signaling pathways underlying resveratrol-mediated protection, from mechanistically distinct types of cellular stress, in the widely used model system for glial cell biology and pharmacology, C6 glioma cells.

We previously showed that in the human neuroblastoma SH-SY5Y cell line UV-irradiation induced caspase activation along with the resulting cleavage of tau [11]. Additionally, SH-SY5Y cells that were UV-irradiated also stained positive with Thioflavin S, indicating the presence of neurofibrillary tangles [11].

Oxidative stress is involved in the pathogenesis of neurodegenerative diseases such as AD [12,13,14]. The brain’s higher susceptibility to oxidative imbalance and increased vulnerability to oxidative damage is likely due to less effective antioxidant systems when compared with other tissues [15]. This susceptibility to damage from oxidative stress likely contributes to the development of AD where higher levels of reactive oxygen species (ROS) were measured during the early stages of the disease affecting neuronal and glial cell types, before pathological hallmarks of AD, such as amyloid plaque formation, can be found [16].

Deoxyribonucleic acid (DNA) damage has been identified as an underlying cause of cellular aging and neurodegenerative disorders such as AD, also affecting neuronal and glial cell types. Markers for DNA damage, particularly oxidative DNA damage, have been found in brain regions affected by AD and in biological fluids of AD patients [17,18]. Additionally, during Mild Cognitive Impairment, a condition potentially preceding AD, oxidative DNA damage is one of the first events associated with neurodegeneration and potentially also with the progression of cognitive impairment ultimately leading to dementia [18]. 

As tau, a microtubule-associated protein primarily expressed by neurons that contributes to the stabilization of microtubules [19] has been a focus of recent such therapy development efforts, the present study has focused extensively on tau protein biochemistry and physiology. A number of kinases phosphorylate tau and tau hyperphosphorylation results in its dissociation from microtubules and subsequently the formation of aggregates, neurofibrillary tangles (NFTs), which are a hallmark of AD [20]. Once polymerized into NFTs, tau loses its function to properly bind tubulin and contribute to the assembly of microtubules. Therefore, blocking the pathological hyperphosphorylation of tau has been deemed a therapeutic target for AD and potentially other tauopathies [21,22]. In addition to tau hyperphosphorylation, a separate pathway, the caspase-mediated cleavage of tau, which produces a cleaved form of tau that assembles into filaments, has been described as contributing to NFT formation and loss of tau function [23,24].

Exposure to ultraviolet (UV) radiation can induce the production of ROS, which in turn can result in oxidative damage and DNA damage [25]. Extracellularly applied chemicals inducing oxidative stress, such as *tert*-butyl hydroperoxide (tBHP), on the other hand have been used extensively to study the effects on calcium dependent signal transduction [26] and cellular viability [27]. 

In the present study, we provide evidence that UV-irradiation of C6 cells leads to the activation of caspases, which in turn results in tau cleavage and NFT formation, a process that can be blocked with the cell-permeable pan-caspase inhibitor *N*-Benzyloxycarbonyl-Val-Ala-Asp(O-Me) fluoromethyl ketone (zVAD-fmk). In addition, we determined that tBHP treatment induced caspase activation along with cleaved tau and NFT formation, as well. For both paradigms of oxidative stress and cell damage, UV-irradiation and tBHP treatment, we identified whether resveratrol was protective in the C6 cell line. Our goal was to identify mechanisms underlying resveratrol’s selective protective action in the nervous system, as a previous report indicates that resveratrol is capable of altering important antioxidant defenses in the C6 cell line [7]. Specifically, we determined how resveratrol acts as a protective agent in C6 cells during oxidative stress and DNA damage, where we found that it prevents tau cleavage and NFT formation induced by both paradigms of oxidative stress and cell damage. However, for each of the experimental paradigms, resveratrol acted at different points in the pathway: while during oxidative stress induced by extracellularly applied tBHP resveratrol controlled caspase activity, during DNA damage resulting from UV-irradiation, resveratrol acted downstream of caspases by regulating the phosphorylation state of tau to exert its protective function.

## 2. Results

### 2.1. Resveratrol Protects C6 Cells against DNA Damage and Oxidative Stress

**Figure 1:**
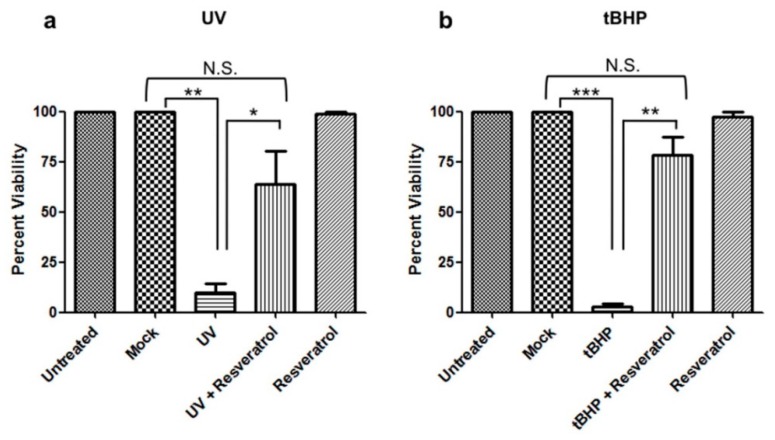
Resveratrol prevents apoptosis induced by UV-irradiation and tBHP treatment in C6 cells. C6 cells were pretreated with resveratrol and either (**a**) UV-irradiated or (**b**) treated with tBHP. Viability was determined 24 h later. C6 cells that were UV-irradiated or treated with tBHP showed a significant decrease (*p* < 0.05) in viability compared to mock and untreated cells. C6 cells pretreated with resveratrol and then (**a**) UV-irradiated or (**b**) tBHP treated showed a significant increase (*p* < 0.05) in viability compared to UV-irradiated or tBHP treated cells alone. (**a**) There was a significant difference in cell viability between mock vs. UV (**, *p* = 0.0026). UV treated cells pretreated with resveratrol showed a significant increase in viability vs. UV treated cells (*, *p* = 0.0354). Mock treated cells compared to UV treated cells pretreated with resveratrol showed no significant difference (Not Significant (N.S.), *p* = 0.0974). (**b**) There was a significant decrease in cell viability in tBHP treated compared to mock treated cells (***, *p* < 0.0001). C6 cells treated with tBHP and pretreated with resveratrol showed a significant increase in cell viability compared to tBHP treated cells (**, *p* = 0.0012). C6 cells treated with tBHP and pretreated with resveratrol showed no significant difference in cell viability compared to mock (N.S., *p* = 0.0758). All experiments were performed in triplicate and values were expressed as mean +/− SEM.

C6 cells were UV-irradiated to induce DNA damage or treated with tBHP to induce oxidative stress, which resulted in apoptotic cell death in both cases (Figure 1a,b, respectively). Pretreatment with resveratrol protected cells against apoptosis induced by either of the two paradigms to induce cell damage (Figure 1, Tables S1 and S2).

### 2.2. UV-Irradiation Induces Caspase Activation and Tau Cleavage in C6 Cells

**Figure 2:**
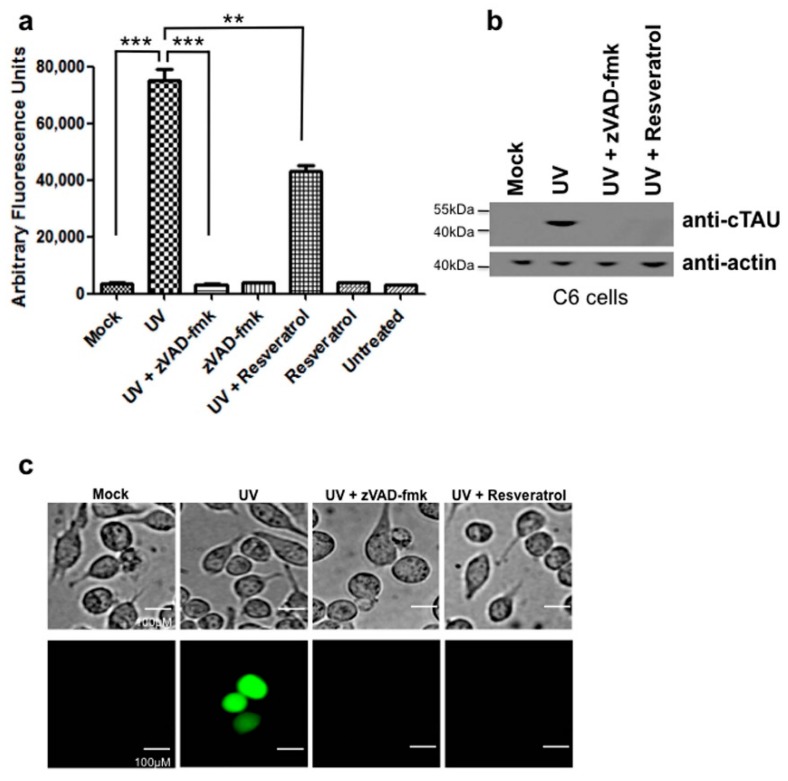
Resveratrol inhibits tau cleavage induced by UV-irradiation in C6 cells. (**a**) C6 cells were UV-irradiated and caspase activity measured using the caspase substrate Ac-DEVD-AFC. Values were plotted as arbitrary fluorescence units. C6 cells that were UV-irradiated showed a significant increase in caspase activity compared to mock treated (***, *p* < 0.0001). C6 cells UV-irradiated and pretreated with resveratrol showed a slight, but significant decrease in caspase activity compared to UV-irradiated cells alone (**, *p* = 0.0015). C6 cells treated with the pan-caspase inhibitor zVAD-FMK showed a significant decrease in caspase activity (***, *p* < 0.0001); (**b**) C6 cells that were UV-irradiated were immunoblotted for cleaved tau. C6 cells that were UV-irradiated showed detectable cleaved tau while cells pretreated with zVAD-fmk or resveratrol showed no detectable cleaved tau; (**c**) Resveratrol prevented NFT formation induced by UV-irradiation in C6 cells. C6 cells that were pretreated with resveratrol were UV-irradiated and then stained with Thioflavin S to detect NFT formation. Scale bar, 100 µm. All experiments were performed in triplicate and values were expressed as mean +/− SEM.

C6 cells were UV-irradiated, harvested after 12 h and assayed for caspase activity. C6 cells that were UV-irradiated had increased levels of caspase 3 activity (Figure 2a). This caspase activity was inhibited by pretreatment with the broad caspase inhibitor zVAD-FMK. Resveratrol significantly reduced caspase activity, but did not return it to baseline levels seen with untreated controls and UV-irradiated, zVAD-FMK treated cells (Figure 2a, Table S3). Subsequently, we measured tau cleavage as a parameter of cellular damage. In the UV-irradiated C6 cells, cleaved tau was detected in UV-irradiated cells, which could be prevented by pretreatment with zVAD-fmk (Figure 2b). Pretreatment with resveratrol also prevented tau cleavage in UV-irradiated C6 cells (Figure 2b) in spite of levels of caspase activity remaining elevated in UV-irradiated cells pretreated with resveratrol (Figure 2a).

### 2.3. UV-Irradiation Causes C6 Cells to Form NFTs, a Process Preventable by Resveratrol Treatment

NFT formation was visualized in C6 cells with Thioflavin-S staining. Cells that were UV-irradiated stained positive for NFTs, while pretreatment with zVAD-fmk to inhibit caspase activation resulted in a lack of staining indicating that NFT formation had been prevented (Figure 2c). C6 cells pretreated with resveratrol also did not stain positive with Thioflavin-S indicating the lack of NFT formation (Figure 2c) even though detectable levels of active caspases were present in these cells.

### 2.4. Oxidative Stress Induces Caspase Activation and Tau Cleavage in C6 Cells, Both of Which are Prevented by Pretreatment with Resveratrol

**Figure 3:**
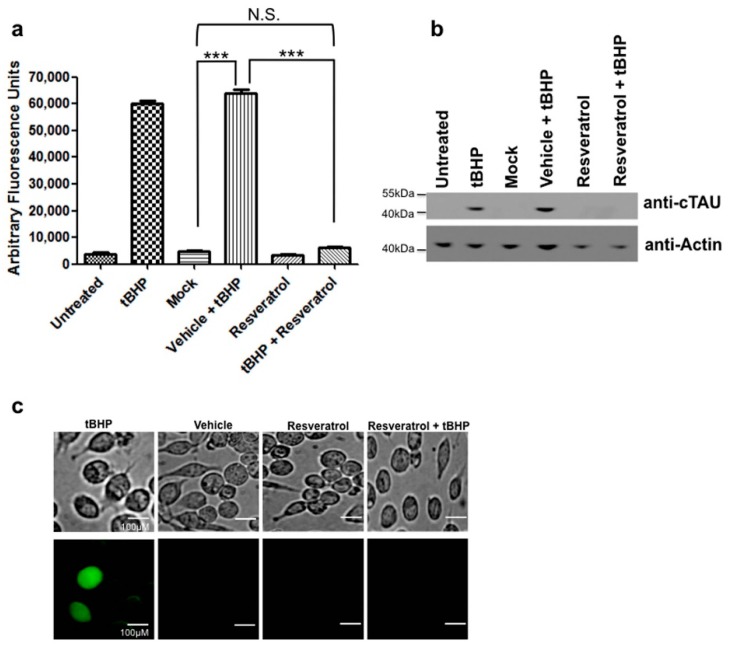
Resveratrol inhibits caspase activation and tau cleavage induced by oxidative stress. (**a**) C6 cells were treated with tBHP to induce oxidative stress and caspase activity measured using the caspase substrate Ac-DEVD-AFC. C6 cells pretreated with resveratrol showed a significant decrease in caspase activity (*p* < 0.05). C6 cells that were treated with tBHP showed a significant increase in caspase activity compared to mock treated (***, *p* < 0.0001). C6 cells treated with tBHP and pretreated with resveratrol showed a significant decrease in caspase activity compared to tBHP treated cells alone (***, *p* < 0.0001). C6 cells treated with tBHP and resveratrol showed no significant difference compared to mock (N.S., *p* = 0.0893); (**b**) C6 cells that were treated with tBHP were immunoblotted for cleaved tau. C6 cells treated with tBHP showed detectable cleaved tau while cells pretreated with resveratrol showed no signs of cleaved tau; (**c**) Resveratrol prevented NFT formation induced by oxidative stress in C6 cells. C6 cells were pretreated with resveratrol and then exposed to tBHP. Thioflavin S staining was performed to detect NFT formation. Scale bar, 100 µm. All experiments were performed in triplicate and values were expressed as mean +/− SEM.

C6 cells that were treated with tBHP to induce oxidative stress showed increased levels of active caspase 3 (Figure 3a, Table S4). When cells were pretreated with resveratrol this tBHP-mediated caspase activation was significantly reduced and indistinguishable from controls (Figure 3a). At the same time, treatment of C6 cells with tBHP also resulted in tau cleavage, i.e., proteolytic truncation of tau at Asp^421^ (Figure 3b). As seen for caspase 3 activation, pretreatment with resveratrol prior to tBHP-mediated induction of oxidative stress also prevented the formation of Asp^421^-truncated tau at detection levels typically achieved with immunoblot analyses (Figure 3b).

### 2.5. Oxidative Stress Leads to NFT Formation in C6 Cells

Treatment of C6 cells with tBHP to induce oxidative stress resulted in the formation of NFTs as detected by Thioflavin-S staining, but after pretreatment with resveratrol, NFTs were not detected with the same cytochemical staining method (Figure 3c).

### 2.6. Resveratrol Mediated Signaling Results in the Hyperphosphorylation of Tau Protein and Prevents Dephosphorylation of Tau at Ser^422^

**Figure 4:**
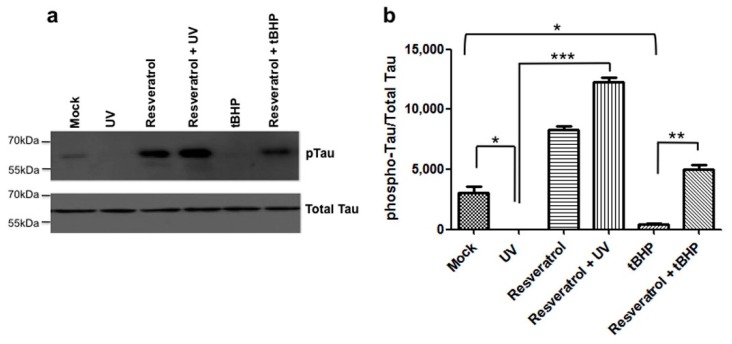
Resveratrol prevents dephosphorylation of tau at Ser^422^. (**a**) C6 cells were pretreated with resveratrol and then exposed to tBHP or UV irradiation. Tau phosphorylation at Ser^422^ was determined immunoblotting and (**b**) quantified; (**b**) Resveratrol significantly (*p* < 0.05) increased tau phosphorylation at Ser^422^ when pretreated prior to UV-irradiation. Resveratrol also increased tau phosphorylation at Ser^422^ when pretreated prior to tBHP treatment, but to a lesser extent than when pretreated prior to UV-irradiation (*p* < 0.05). UV-irradiated C6 cells showed a significant decrease in tau phosphorylation compared to mock treated cells (*, *p* = 0.0347). C6 cells that were UV-irradiated and pretreated with resveratrol showed a significant increase in tau phosphorylation compared to UV-irradiated c6 cells alone (***, *p* = 0.0009). C6 cells treated with tBHP showed a significant decrease in tau phosphorylation compared to mock (*, *p* = 0.0471). C6 cells treated with tBHP and pretreated with resveratrol showed a significant increase in tau phosphorylation compared to tBHP treated cells (**, *p* = 0.0049). All experiments were performed in triplicate and values were expressed as mean +/− SEM.

Tau is phosphorylated at Ser^422^ under control conditions in C6 cells (Figure 4a). When C6 cells undergo DNA damage due to UV-irradiation or are exposed to tBHP-mediated oxidative stress, tau becomes dephosphorylated at Ser^422^ significantly compared to mock treated controls (Figure 4). Pretreatment of C6 cells with resveratrol prevented tau dephosphorylation at Ser^422^ for both paradigms of cell damage (Figure 4) and treatment with resveratrol alone in the absence of UV or oxidative stress-mediated damage increased the levels of tau phosphorylation at Ser^422^ as compared to controls (Figure 4). When C6 cells were pretreated with resveratrol prior to UV-irradiation, tau phosphorylation at Ser^422^ increased to significantly greater levels compared to UV-irradiated or mock treated C6 cells. In addition, when C6 cells were pretreated with resveratrol before exposure to tBHP, phosphorylation of tau at Ser^422^ was significantly increased compared to tBHP treatment alone (Figure 4b). However, unlike the increase in tau phosphorylation observed with resveratrol pretreatment combined with subsequent UV-irradiation, the increase was not significantly greater than mock or resveratrol treatment alone (Figure 4b, Table S5).

## 3. Discussion

Glial cells play an important role in neurodegenerative diseases, but far less is known about underlying mechanisms when compared to well-researched neuronal cells. However, several lines of evidence have implicated glial cells mechanistically in the pathogenesis of neurodegenerative diseases. Previous work by Forster and colleagues demonstrated the presence of tau aggregates in glial cells [28]. In addition, caspase 3 activation has been observed in glial cells in the AD brain [29]. While initially NFTs, as the name suggests, were thought to develop only in neuronal cells, histological studies of brain tissue from AD patients revealed tau-immunoreactive NFTs also in glial cells [30]. With the increasing recognition of the importance of astrocytes as key regulators of nervous system function, identifying their physiological function and pharmacological properties becomes more important. However, their isolation from the brain is a variable, time consuming, and costly procedure, while the C6 glioma cell line has all advantages of a cell line and has been extensively characterized. In addition, resveratrol protects C6 cells against hydrogen peroxide induced oxidative stress [7,8] and can attenuate oxidation-induced DNA damage in C6 glioma cells [17,18]. Furthermore, in C6 rat astroglioma cells, exposure to the Aβ peptide reduced cell growth, while resveratrol pretreatment protected from this toxicity [4]. Resveratrol suppresses interleukin-6 production in astrocyte cultures that had been deprived of glucose and oxygen [31] and reduces neuronal cell death in vivo in a gerbil model of global cerebral ischemia [32].

Astroglia contribute significantly to the regulation of extracellular glutamate levels, a process critical for the prevention of excitotoxic injury [33]. This is of increasing importance, as altered levels of extracellular glutamate have been implicated in neurodegenerative disorders such as Alzheimer’s disease and amyotrophic lateral sclerosis [33]. In addition, increased level of ROS resulting from neurodegenerative diseases are associated with both increased release and decreased uptake of glutamate [34]. Dos Santos and colleagues measured the modulation of glutamate uptake by resveratrol in C6 cells [35]. In this study, the regulatory effect of resveratrol was highly concentration dependent. For example, when C6 cells were treated with 100 μM resveratrol glutamate uptake increased by 50%, but when the C6 cells were treated with a higher resveratrol concentration (250 μM) the increase was only 30%. This significant difference was attributed to a biphasic effect of resveratrol [35]. Similar biphasic effects resulting from different resveratrol concentrations were observed in the same study with respect to glutamine synthetase activity and cellular viability: while 100 μM resveratrol increased glutamine synthetase activity, no effect on this enzyme was seen at 250 μM resveratrol. Cellular apoptosis was measured with the lactate dehydrogenase release assay and was not observed at 100 μM resveratrol, while significant lactate dehydrogenase release was seen when C6 cells had been treated with 250 μM resveratrol, which was also accompanied by propidium iodide uptake as a measure of cell death at this concentration of resveratrol [35]. This example of the pleiotropic effects of resveratrol and their concentration dependence even within the same highly reproducible and well-characterized in vitro model of glia cell function, which was also used in the present study, illustrates the high specificity and sometimes antagonism of cellular mechanisms such as the activation of anti- versus pro-oxidant or anti- versus pro-apoptotic pathways modulated by resveratrol, and their dependence on the resveratrol concentrations and cell type.

Extending these observations in the present study, we identified in a glial cell line that, like neurons, caspase activation leading to proteolytic cleavage of tau resulted in the formation of NFTs. This first report linking the activation of caspases induced by either oxidative stress or DNA damage and the cleavage of tau leading to NFT formation in glial cells contributions to the notion of a significant glial contribution to neurodegeneration. The activation of caspases and cleavage of key cellular proteins may contribute to injury and damage of glia in the AD brain potentially resulting in loss of function. NFTs in glial cells, as seen in AD [30] and in the present study using controlled in vitro paradigms of cellular damage (Figure 2 and Figure 3), potentially contribute to pathological processes and disease pathogenesis like in neurons.

**Figure 5:**
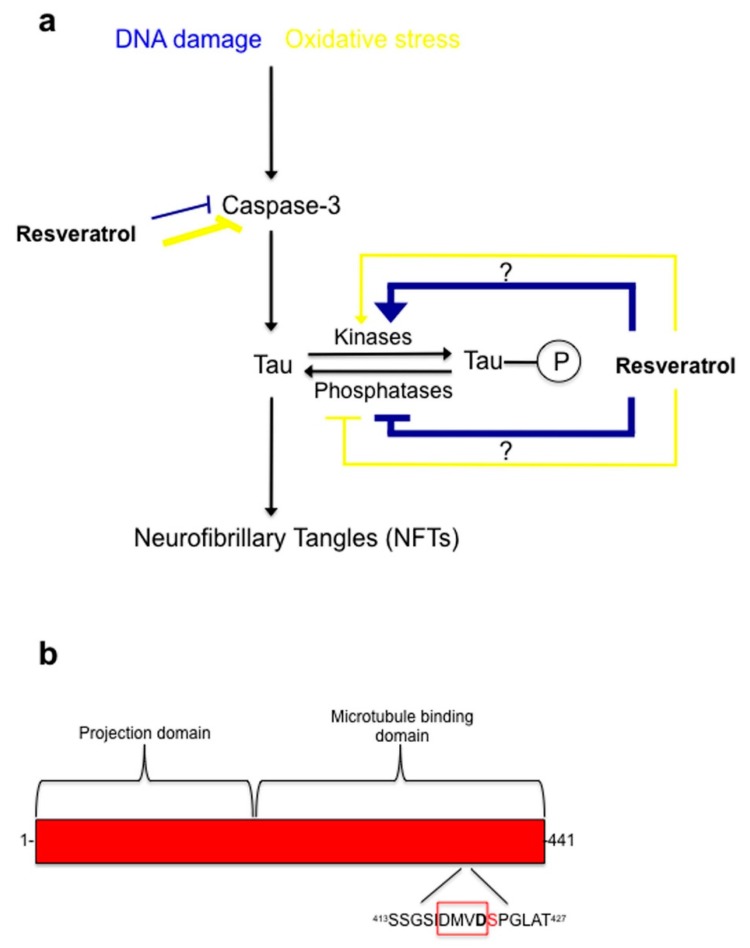
Proposed model of resveratrol mode of action during oxidative stress and DNA damage in C6 cells. (**a**) During oxidative stress and DNA damage resveratrol protective properties involves inhibition of caspase-3 activity. Resveratrol does this potently (yellow thick line) during oxidative stress, but during DNA damage it is less effective (blue thin line) at inhibiting caspase-3 activity. Additionally, resveratrol works on the phosphorylation state of tau at Serine residue 422 by regulating a kinase(s) or phosphatase(s). Resveratrol works effectively (blue thick line) during DNA damage by hyper-phosphorylating tau at Serine 422, which is directly next to the caspase-3 cleavage site, Asp 421. This could potentially block the caspase cleavage site from being available for caspase-3. Resveratrol is less effective (yellow thin line) at regulating tau phosphorylation at Ser^422^ during oxidative stress; (**b**) Diagram of human tau showing the caspase-3 cleavage site (Asp^421^) and phosphorylation site (Ser^422^). DMVD is a peptide sequence caspases recognize on their substrates, with caspase-mediated protein/peptide cleavage occurring between Asp^421^ and Ser^422^.

The present study also provided evidence for a protective effect of resveratrol in C6 cells during oxidative stress and DNA damage. Treatment of C6 cells with resveratrol prevented apoptosis induced by UV-irradiation and the resulting DNA damage or tBHP treatment and the concomitant oxidative stress (Figure 1). In addition, resveratrol was able to prevent tau cleavage, specifically the formation of Asp^421^-truncated tau, and NFT formation induced by both paradigms of cellular damage (Figure 2 and Figure 3). Interestingly, although resveratrol was protective against both treatments, its mode of action varied. For DNA damage induced by UV-irradiation, resveratrol acted downstream of caspases, at the level of tau phosphorylation (Figure 2), while resveratrol worked at the level of caspase activation when oxidative stress was induced by tBHP (Figure 3 and Figure 5). 

The mechanisms underlying the resveratrol’s protective effect against UV-induced cell damage are unknown. We show for the first time that one target for resveratrol during DNA damage induced by UV-irradiation is the microtubule associated protein tau. Specifically, levels of tau phosphorylation, specifically at Serine 422, a critical residue situated immediately adjacent to the caspase 3 cleavage site (Asp^421^) were reduced for both paradigms of cellular damage (Figure 4). Phosphorylation of tau protein at this residue may be protective by preventing caspases from accessing the adjacent cleavage site. When tau is dephosphorylated at Ser^422^ the resulting exposed caspase cleavage site could allow the caspase-mediated cleavage of tau and formation of Asp^421^-truncated tau. Furthermore, the present study provides evidence that resveratrol increases levels of tau phosphorylation at Serine 422, thereby potentially preventing caspase cleavage of tau in line with the reasoning that tau dephosphorylated at Ser^422^ is more susceptible to caspase-mediated cleavage. In both experimental paradigms of cellular damage, oxidative stress (Figure 3) and DNA damage (Figure 2), resveratrol was found to be protective, by fully or partially eliminating caspase activation, respectively.

For oxidative stress induced by tBHP treatment, we show for the first time that in C6 cells resveratrol is protective and prevents apoptosis by directly inhibiting caspase 3 activity resulting in reduced formation of Asp^421^-truncated tau and of NFTs, and to a lesser extent by inducing tau phosphorylation at Ser^422^ (Figure 5). On the other hand, for DNA damage induced by UV-irradiation, resveratrol’s protective action is potentially mediated to a larger extent by increased phosphorylation of tau protein at Ser^422^, preventing subsequent tau cleavage than by direct inhibition of caspase 3 activity.

In conclusion, we provide experimental evidence for resveratrol being effective at inhibiting apoptosis induced by distinct noxious stimuli and for its action at different steps in the signaling pathway leading to NFT formation depending on the initial stimulus producing cell damage. While the identification of such pleiotropic activity adds to the notion of resveratrol as a potentially therapeutically relevant compound, future studies might identify specific resveratrol binding partners responsible for the activation of protective mechanisms depending on the respective stimulus, inducing neurodegeneration.

## 4. Methods

### 4.1. Cell Culture

The C6 glial cell line was obtained from the American Type Culture Collection (ATCC, Rockville, MD, USA). The cells were seeded in flasks and cultured in F-12K media supplemented with 5% Fetal Bovine Serum (FBS) and 15% horse serum (ATCC). Cells were kept at 37 °C in an atmosphere of 95% air and 5% CO_2_. 

### 4.2. Resveratrol and tBHP Treatment and Assessment of Cell Viability

For induction of oxidative stress, cells were treated overnight with media containing 50 µM *t*BHP and using water as a vehicle control. For experiments involving resveratrol-mediated protection, cells were pretreated with 100 µM resveratrol (or DMSO as a vehicle control) for 2 h prior to the addition of tBHP. Cell viability was determined using the Trypan blue viability test.

### 4.3. Caspase Activity Assay

To measure caspase activity in C6 cells, cells were detached enzymatically and collected 12 h after treatment and subjected to centrifugation at 2000× *g*. The pellets were resuspended in caspase buffer (20 mM HEPES, pH 7.5, 50 mM KCl, 1.5 mM MgCl_2_, 1 mM EDTA, 1 mM EGTA, 1 mM DTT supplemented with protease inhibitor cocktail (Complete ULTRA Tablets, Roche Diagnostics, Indianapolis, IN, USA), and lysed with three freeze/thaw cycles. The lysates were then incubated for one hour at 37 °C with 0.2 μM fluorogenic caspase-3 substrate, Ac-DEVD-AFC (Ac-Asp-Glu-Val-Asp-7-Amino-4-trifluoromethylcoumarin, Santa Cruz Biotechnology, Dallas, TX, USA; product number, sc-311274). The amount of the fluorescent product resulting from caspase-3 activity was determined fluorometrically (excitation 405 nm, emission 535 nm) and activity was expressed in relative arbitrary fluorescence units as described by us previously [11]. 

### 4.4. Antibodies

Mouse anti-tau, caspase cleaved (truncated at Asp^421^) antibody (MilliporeSigma, Billerica, MA, USA; product number, MAB5430) was used at a dilution of 1:1000 to detect cleaved tau. Rabbit anti-tau (phospho-Ser^422^) (GenScript, Piscataway, NJ, USA; product number, A00900) was used at a dilution of 1:1000 to detect tau phosphorylation at Ser^422^. Full length tau was detected using the tau 5A6 antibody (Developmental Studies Hybridoma Bank, Iowa City, IA, USA; product number, 5A6) at a dilution of 1:500. Mouse anti-Actin (MilliporeSigma; product number, MAB1501R) was used at a dilution of 1:1000 to detect actin as a loading control in Western blotting assays, as described by us previously [11].

### 4.5. Thioflavin S Staining

To detect NFT formation in C6 cells, cells were rinsed in distilled water and fixed with 3% PFA for 5 min at room temperature. Cells were then washed 3 times for 5 min each with PBS and permeabilized with 0.2% Triton-X-100 for 3 min at room temperature. Cells were then washed 3 times in PBS for 5 min each and incubated with Thioflavin S (Sigma-Aldrich, St. Louis, MO, USA; product number, T1892) at a concentration of 0.05% in distilled water for 5 min. After 5 min, the C6 cells were washed in 70% ethanol for 5 min, and rinsed subsequently with distilled water 10 times for 5 min each, followed by an overnight wash, as described by us previously [11].

### 4.6. UV-Irradiation of Cells

C6 cells were plated in a 6-well cell culture plate (TPP 35 mm tissue culture plate, MidSci, St. Louis, MO, USA) and allowed to adhere overnight. The following day, cells were treated with the pan-caspase inhibitor zVAD-fmk (FMK001, R&D systems, Minneapolis, MN, USA) at a concentration 100 µM or with 100 µM of resveratrol. Two hours after pretreatment, cells were UV-irradiated (UV-C, 254 nm) for 5 min using a 3UV transilluminator (BioChemi System, UVP, Upland, CA, USA) as described by us previously [18]. 12 h after UV-irradiation, cells were harvested for immunoblot analysis, caspase activity measurements or stained for NFT formation using Thioflavin S.

### 4.7. Data Analysis and Statistics

For statistical analysis Prism5 software (GraphPad Inc., La Jolla, CA, USA) was used. Densitometric analysis of Western blotting experiments was performed using ImageJ software (Version 1.50i, Developer: Wayne Rasband, National Institutes of Health, Bethesda, MA, USA) to quantify individual bands. All values are expressed as mean +/− standard error of the mean (SEM). Statistical comparison of means was performed by Student’s *t*-test for comparisons between two groups or by one-way analysis of variance (ANOVA) and the Bonferroni post-hoc test, for multiple comparisons with pre-treatment conditions (untreated, mock, or resveratrol treated) and insults (tBHP, UV-irradiation) as variables. Statistical significance was defined as *p* < 0.05. Using the Tukey post-hoc test produced the same results as using the Bonferroni post-hoc test.

## Supplementary Materials

Supplementary materials can be found at www.mdpi.com/1422-0067/18/7/1521/s1.
