# Supplementary material for: Distinct Mechanisms Underlying Resveratrol-Mediated Protection from Types of Cellular Stress in C6 Glioma Cells
Source: Int J Mol Sci. 2017 Jul 14;18(7):1521. doi: 10.3390/ijms18071521 (PMC5536011; doi:10.3390/ijms18071521)
Supplement: Supplementary file 1 [file ijms-18-01521-s001.pdf]

Table S1. One way ANOVA comparison of the viability of UV irradiated C6 cells in the presence or absence of resveratrol

Comparison	Significant? P < 0.05?
Untreated vs Mock	No
Untreated vs UV	Yes
Untreated vs UV + Resveratrol	No
Untreated vs Resveratrol	No
Mock vs UV	Yes
Mock vs UV + Resveratrol	No
Mock vs Resveratrol	No
UV vs UV + Resveratrol	Yes
UV vs Resveratrol	Yes
UV + Resveratrol vs Resveratrol	No

R^2 0.9335, F 28.06

	**df**
Treatment	4
Individual	2
Residual	8
Total	14

Table S2. One way ANOVA comparison of the viability of tBHP treated C6 cells in the presence or absence of resveratrol

Comparison	Significant? P < 0.05?
Untreated vs Mock	No
Untreated vs tBHP	Yes
Untreated vs tBHP + Resveratrol	No
Untreated vs Resveratrol	No
Mock vs tBHP	Yes
Mock vs tBHP + Resveratrol	No
Mock vs Resveratrol	No
tBHP vs tBHP + Resveratrol	Yes
tBHP vs Resveratrol	Yes
tBHP + Resveratrol vs Resveratrol	No

R^2 0.9810, F 103.4

	**df**
Treatment	4
Individual	2
Residual	8
Total	14

Table S3. One way ANOVA comparison of caspase activation induced by UV irradiation in the presence or absence of resveratrol

Comparison	Significant? P < 0.05?
Mock vs UV	Yes
Mock vs UV + zVAD-fmk	No
Mock vs zVAD-fmk	No
Mock vs UV + Resveratrol	Yes
Mock vs Resveratrol	No
Mock vs Untreated	No
UV vs UV + zVAD-fmk	Yes
UV vs zVAD-fmk	Yes
UV vs UV + Resveratrol	Yes
UV vs Resveratrol	Yes
UV vs Untreated	Yes
UV + zVAD-fmk vs zVAD-fmk	No
UV + zVAD-fmk vs UV + Resveratrol	Yes
UV + zVAD-fmk vs Resveratrol	No
UV + zVAD-fmk vs Untreated	No
zVAD-fmk vs UV + Resveratrol	Yes
zVAD-fmk vs Resveratrol	No
zVAD-fmk vs Untreated	No
UV + Resveratrol vs Resveratrol	Yes
UV + Resveratrol vs Untreated	Yes
Resveratrol vs Untreated	No

R^2 0.9935, F 303.9

	df
Treatment	6
Individual	2
Residual	12
Total	20

Table S4. One way ANOVA comparison of caspase activation induced by tBHP in the presence or absence of resveratrol

Comparison	Significant? P < 0.05?
Untreated vs tBHP	Yes
Untreated vs Mock	No
Untreated vs Vehicle + tBHP	Yes
Untreated vs Resveratrol	No
Untreated vs tBHP + Resveratrol	No
tBHP vs Mock	Yes
tBHP vs Vehicle + tBHP	No
tBHP vs Resveratrol	Yes
tBHP vs tBHP + Resveratrol	Yes
Mock vs Vehicle + tBHP	Yes
Mock vs Resveratrol	No
Mock vs tBHP + Resveratrol	No
Vehicle + tBHP vs Resveratrol	Yes
Vehicle + tBHP vs tBHP + Resveratrol	Yes
Resveratrol vs tBHP + Resveratrol	No

R^2 0.9992, F 2432

	df
Treatment	5
Individual	2
Residual	10
Total	17

Table S5. One way ANOVA comparison of phosphorylated Tau in C6 cells UV irradiated or treated with tBHP in the presence or absence of resveratrol

Comparison	Significant? $P < 0.05$?
Mock vs UV	Yes
Mock vs Resveratrol	Yes
Mock vs Resveratrol + UV	Yes
Mock vs tBHP	Yes
Mock vs Resveratrol + tBHP	Yes
UV vs Resveratrol	Yes
UV vs Resveratrol + UV	Yes
UV vs tBHP	No
UV vs Resveratrol + tBHP	Yes
Resveratrol vs Resveratrol + UV	Yes
Resveratrol vs tBHP	Yes
Resveratrol vs Resveratrol + tBHP	Yes
Resveratrol + UV vs tBHP	Yes
Resveratrol + UV vs Resveratrol + tBHP	Yes
tBHP vs Resveratrol + tBHP	Yes

R^2 0.9949, F 391.3

	df
Treatment	5
Individual	2
Residual	10
Total	17
